# Transcriptional regulation of the potential tumor suppressor *ABI3* gene in thyroid carcinomas: interplay between methylation and *NKX2-1* availability

**DOI:** 10.18632/oncotarget.8416

**Published:** 2016-03-27

**Authors:** Lais Moraes, Ana Luiza R. Galrão, Ileana Rubió, Janete M. Cerutti

**Affiliations:** ^1^ Genetic Bases of Thyroid Tumors Laboratory, Division of Genetics, Department of Morphology and Genetics, Escola Paulista de Medicina, Universidade Federal de São Paulo, SP, Brazil; ^2^ Department of Biological Sciences, Universidade Federal de São Paulo, SP, Brazil

**Keywords:** ABI3, NKX2-1, DNA methylation, follicular thyroid carcinoma, cancer-specific differentially methylated region (cDMR)

## Abstract

We previously reported that *ABI3* expression was decreased in thyroid cancer tissues and that ectopic expression of *ABI3* in a follicular thyroid carcinoma cell line delayed cell cycle progression and inhibited cell proliferation, invasion, migration and tumor formation in athymic mice. These data indicated that *ABI3* is a tumor suppressor gene; however the mechanism through which *ABI3* is silenced in thyroid carcinomas is unknown. We here show that treatment of four follicular thyroid carcinoma cell lines with 5-aza-dC induced demethylation of a specific region of the *ABI3* promoter and restored *ABI3* expression. In contrast, 5-aza-dC treatment did not restore *ABI3* expression in a non-thyroid cell line, suggesting a tissue-specific regulation. We additionally show that 8 CpG sites located within the *ABI3* promoter are hypermethylated in most thyroid carcinoma samples and the degree of methylation correlated with *ABI3* expression. Narrowing the region to specific CpG sites, the CpG_4-6_ sites showed the largest difference between benign and malignant lesions. *In silico* analysis revealed that these CpG sites flank a canonical binding site for NKX2-1, a thyroid specific transcriptional factor. Analysis of thyroid samples shows a correlation between *NKX2*-*1* and *ABI3* expression. *In vitro* assays demonstrate that NKX2-1 was required for ABI3 expression. Luciferase assay further confirmed the promoter activity of this region, which was increased when the cells were co-transfected with NKX2-1. Our study shows that the transcriptional silencing of *ABI3* in cancer cells occurs via methylation and uncovered a previously unrecognized role for NKX2-1 in the regulation of *ABI3*.

## INTRODUCTION

We previously reported that *ABI3* expression is reduced or lost in follicular cell-derived thyroid carcinomas as compared to normal tissues and follicular thyroid adenomas (FTA) [1]. We further demonstrated that ectopic expression of *ABI3* inhibited cell proliferation, invasion, migration and delayed cell cycle progression of thyroid carcinoma cell line *in vitro*. Moreover, *ABI3* expression inhibited tumor formation in athymic mice [1]. These findings provide evidences that *ABI3* is a tumor suppressor gene that plays important roles in the malignant transformation of thyroid tumors.

In addition to its tumor suppressive effect, it has been proposed that *ABI3* is involved in tumor progression. Loss of *ABI3* expression was reported in several cancer cell lines, including a highly metastatic U87 human glioma cell line. The authors further showed that forced expression of *ABI3* into U87 cells suppressed cell motility and metastatic dissemination *in vivo* [2].

ABI3, like ABI1 and ABI2, which promote the Abl-mediated phosphorylation of MENA and WAVE2, is present in a macromolecular WAVE complex (Abi1/Abi2, Sra1/cyfip1, Nap1, HSPC300 and WAVE/Scar). Nevertheless, it is likely to play a different role in the regulation of Abl [3]. It has been suggested that ABI3 interact with the SH3 domain of the insulin receptor substrate protein 53 (IRSp53), a WAVE2-binding protein that is not included in the aforementioned protein complex. Therefore, ABI3 might compete with WAVE2 for binding to IRSp53 [4]. These findings indicate that ABI3 interacts via SH3 domain with different proteins in a context-dependent manner and they are someway involved in cytoskeletal reorganization.

More extensive studies are needed to identify proteins that may interact with ABI3 in thyroid cells and, particularly, to identify the underlying mechanism by which *ABI3* expression is lost in follicular cell-derived thyroid cancer and carcinoma cell lines.

In this paper, we focus on the mechanism associated with ABI3 silencing in thyroid carcinomas. It is recognized that DNA methylation is the main mechanism linked with gene expression control [5]. DNA methylation typically occurs at cytosines in cytosine-guanine dinucleotides (CpG), which are randomly distributed through the genome. CpG sites tend to occur in cluster called CpG islands. Nearly 70% of annotated gene promoters are associated with CpG islands, which typically remain unmethylated in normal cells [6].

One study, through comparison of global methylation profile of different chronic lymphocytic leukemia prognostic subgroups, reported that *ABI3* was silenced via DNA hypermethylation [7]. The authors found a high degree of methylation at CpG sites located within intron 1 of *ABI3* gene in the samples from the poor-prognosis group compared with that seen in the samples from favorable prognosis group [7].

We here speculate whether decrease or absence of *ABI*3 expression is correlated with hypermethylation of the *ABI3* in primary follicular thyroid carcinomas (FTC) tissues and in follicular thyroid carcinoma cell lines.

We here demonstrated that *ABI3* expression was restored in four thyroid carcinoma cells (FTC 238, FTC 236, FTC 133 and WRO) after treatment with demethylating agent 5-aza-dC. We identified a cancer-specific differentially methylated region located in the *ABI3* promoter, which is hypermethylated in thyroid cell lines and thyroid carcinoma samples while is hypomethylated in the benign samples (FTA) and in a non-thyroid cell model (melanoma cells). Moreover, we show that the regulatory function of this differentially methylated region might be dependent on the expression of *NKX2-1,* also named thyroid specific transcription factor 1. The results indicate that promoter methylation plays an important role in the down-regulation of ABI3 expression in thyroid cell lines and thyroid carcinoma tissues and explain, at least in part, why *ABI3* silencing might occur in a cancer- and tissue-specific manner.

## RESULTS

### *ABI3* expression was restored by 5-aza-dC treatment in thyroid carcinoma cell lines

**Figure 1:**
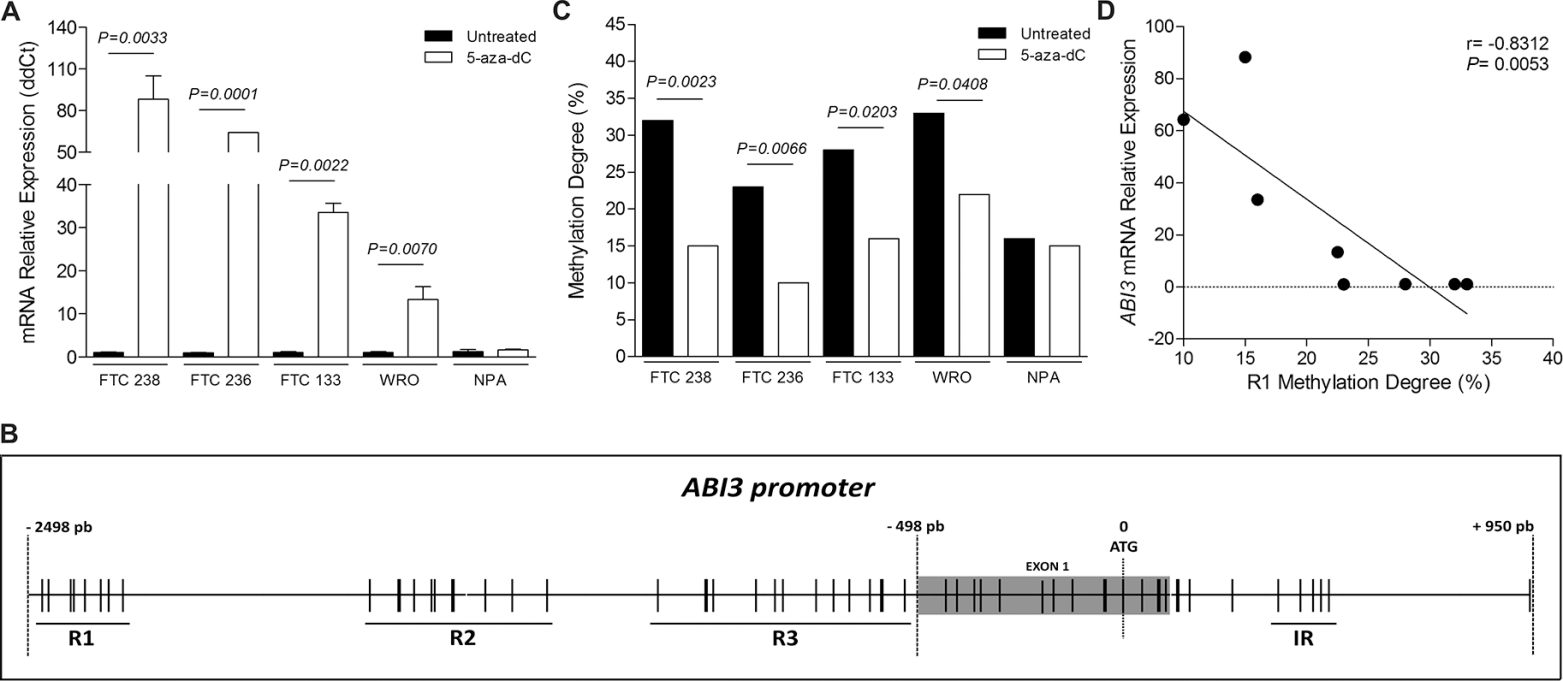
(**A**) *ABI3* expression in follicular thyroid carcinoma cell lines (FTC 238, FTC 236, FTC 133 and WRO) and melanoma cell line (NPA) following treatment with 5-aza-dC. The expression of *ABI3* was restored after 5-aza-dC treatment. No effect on the expression of *ABI3* was observed in melanoma cells (NPA). (**B**) Representative illustration of CpG sites (black lines) in *ABI3* promoter (−498 to −2498 bp relative to ATG). The multiple adjacent CpG sites of each region (R1, R2 and R3) evaluated in the present study are underlined. The CpG sites located in the intronic region (IR), previously described as methylated in leukaemia, is showed. (**C**) Thyroid carcinomas cells were demethylated following 5-aza-dC treated, while no effect was observed in melanoma cells (**D**) Methylation degree of R1 negatively correlated with the *ABI3* expression in follicular thyroid carcinoma cells.

To identify whether the transcriptional repression of *ABI3* in thyroid carcinomas results from DNA methylation in the promoter region of the gene, *ABI3* expression was assayed in thyroid follicular carcinoma cell lines (FTC 238, FTC 236, FTC 133 and WRO) following treatment with the demethylating agent 5-aza-2′-deoxycitidine (5-aza-dC). The incubation with 5-aza-dC resulted in a significant increase of *ABI3* mRNA in FTC 238 (88.20 ± 16.85-fold; *P* = 0.0033), FTC 236 (64.10 ± 0.10-fold; *P* = 0.0001), FTC 133 (33.55 ± 2.15-fold; *P* = 0.0022) and WRO (13.37 ± 2.95-fold, *P* = 0.0070), compared to untreated cells (Figure 1A). Restoration of *ABI3* expression in thyroid carcinoma cells by 5-aza-dC treatment confirmed a causal correlation between DNA hypermethylation and *ABI3* silencing in thyroid cell lines.

We also analyzed whether the transcription level of *ABI3* is regulated by DNA methylation in a melanoma cell line (NPA). No obvious change was seen in ABI3 expression after the treatment of NPA cells with 5-aza-dC (Figure 1A). Together, this data suggests that the hypermethylation and *ABI3* silencing might occurs in a tissue specific-manner.

### Identification of a new cancer-specific differentially methylated region (cDMR) within *ABI3* promoter

Using the criteria of CpG islands described by Gardiner-Garden and Frommer and revised by Takai and Jones, no CpG island was found between position −10,000 pb and +500 pb relative to ATG of *ABI3* gene. The status of five previously described CpG sites located within intron 1 (Intronic Region, IR), between positions +360 to +481 of *ABI3* gene, were analyzed in WRO, FTC 238 and NPA cells (Figure 1B) [7].

The methylation status of each CpG was obtained by sequencing PCR clones from bisulfite treated genomic DNA purified from 5-aza-dC-treated and untreated cells. Although the five CpG sites were methylated at different degree, there was no difference in the overall degree of methylation at these sites between untreated and 5-aza-dC treated thyroid cell lines (WRO, FTC 238) and melanoma cell (NPA cells) (Supplementary Figure S1A).

In a next step, we focus on the methylation status of the ABI3 promoter region, from −2498 pb to −498 pb relative to ATG of *ABI3* gene (NC_000017.11, NM_016428.2). Thirty-three CpG sites around the transcriptional start site were identified. Eight CpG sites from −2467 pb to −2285 pb (Region 1, R1); 11 CpG sites from −1708 pb to −1284 pb (Region 2; R2); and 14 CpG sites from −1089 pb to −509 pb (Region 3; R3) (Figure 1B).

The methylation pattern of each CpG across 3 regions of the *ABI3* promoter was determined for each cell line. Although the CpG sites located at R2 and R3 regions were partially methylated, having a combination of methylated and unmethylated sites, the examining of the methylation degree of each region for each cell line revealed no difference between untreated and 5-aza-dC treated thyroid cell lines (WRO, FTC 238) and melanoma cell (NPA cells) (Supplementary Figure S1B and S1C). These findings indicate that these CpG sites are not likely to be methylated/demethylated in thyroid tumors.

The bisulfite sequencing of the 8 CpG sites located at R1 region revealed higher methylation degree of these sites in untreated thyroid follicular carcinoma cells as compared to 5-aza-dC treated FTC 238 (*P* = 0.0023) and WRO (*P* = 0.0408) cells (Figure 1C). We then evaluated the methylation status of CpG sites located at R1 region in two additional thyroid carcinoma cell lines (FTC 236 and FTC 133), which the expression of ABI3 was increased following treatment with 5-aza-dC (Figure 1A). These CpG sites were highly methylated in untreated cells as compared to 5-aza-dC treated FTC 236 (*P* = 0.0066) and FTC133 (*P* = 0.0203) cells. Low methylation degree was observed in these sites in both untreated (16%) and 5-aza-dC treated (15%) melanoma cells (Figure 1C).

As illustrated in Figure 1D, the methylation degree of CpG sites located at R1 correlated negatively with *ABI3* expression in thyroid follicular cancer cells (*r* = −0.8312 and *P* = 0.0053), perhaps characterizing a new cDMR.

### Screening of the cancer-specific differentially methylation region localized within *ABI3* promoter in thyroid carcinomas samples

**Figure 2:**
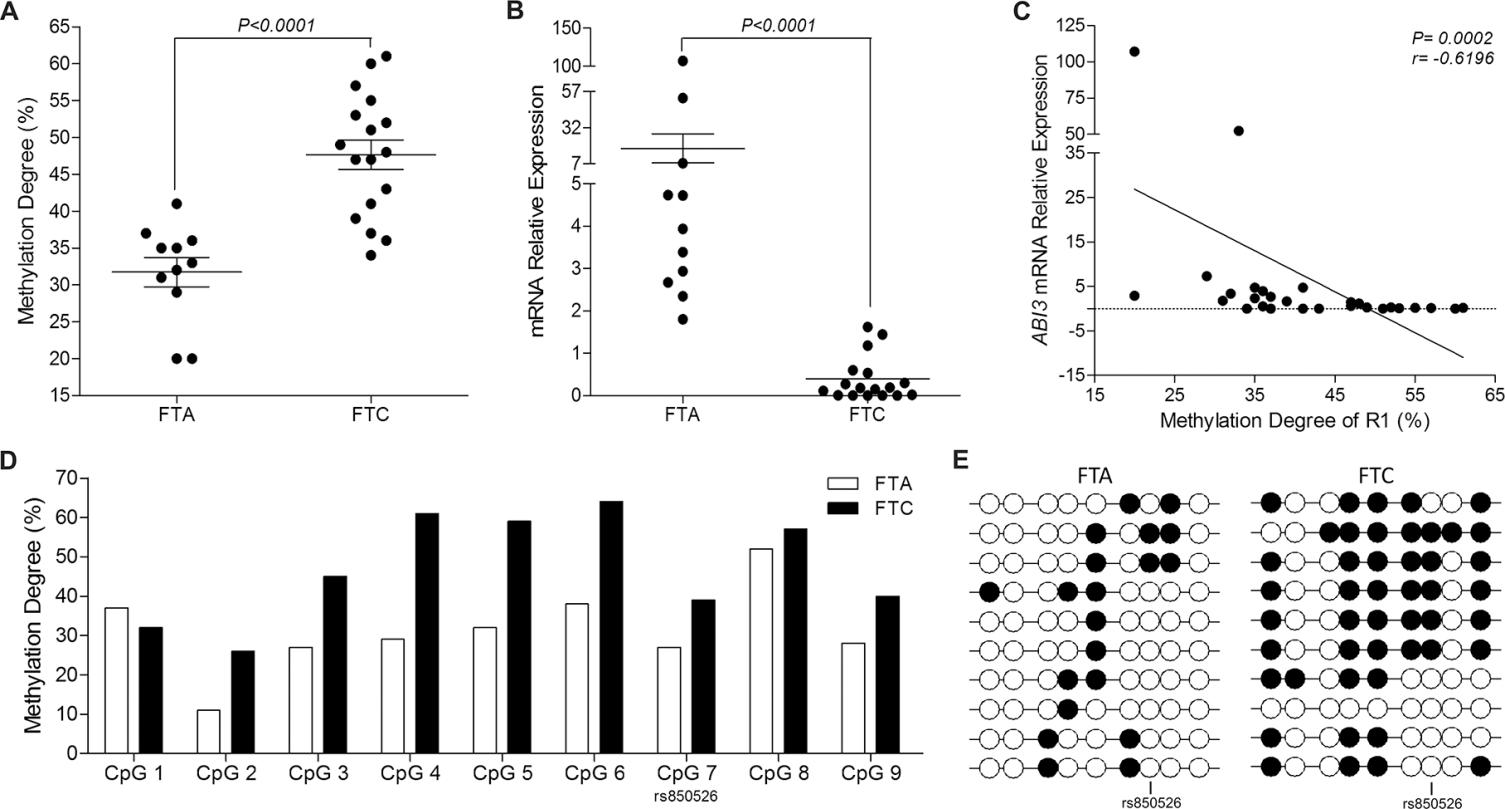
*ABI3* expression and methylation analysis in thyroid tumor samples (**A**) The R1 was hypermethylated in FTC samples compared to FTA. (**B**) *ABI3* expression is absent in most FTC, while is expressed at high levels in FTA. (**C**) A relatively strong negative correlation is observed between *ABI3* expression and R1 methylation in both FTA and FTC. (**D**) Significant differences were found for each CpG site within the R1. The largest differences were observed for CpG_4–6_, which were hypermethylated in FTCs compared to FTAs. CpG_7_ corresponds to SNP rs850526. (**E**) Representative results of the bisulfite sequencing of the 10 selected clones evaluated for a single patient with FTA and one patient with FTC. FTA: follicular thyroid adenoma; FTC: follicular thyroid carcinoma.

To investigate whether the down-regulation of *ABI3* expression in FTC, as compared to FTA, results from hypermethylation of the CpG sites located in the promoter region of *ABI3* gene, we focused our analysis of DNA methylation on a region within the promoter (R1) that was methylated in thyroid carcinoma cell lines. Bisulfite sequencing method was used to analyze the methylation level of the CpG sites located at the R1 in 11 FTA and 17 FTC samples. The mean level of methylation in all 8 CpG sites was higher in FTC (47.65%) compared to FTA (31.73%) (*P* < 0.0001) (Figure 2A).

To test whether *ABI3* transcription is associated with DNA methylation of its promoter, we used qPCR to assess the expression levels of *ABI3* in thyroid samples and correlated with methylation status. *ABI3* expression was lost in most FTCs (mean ± SD 0.3882 ± 0.12) as compared to FTA (mean ± SD 17.58 ± 9.990; *P* < 0.0001) (Figure 2B). When methylation degree and *ABI3* expression in thyroid tissues was compared, we found that the expression of *ABI3* mRNA decreased in most FTC samples in which *ABI3* hypermethylation was observed. In fact, we show a strong negative correlation between methylation degree of the CpG sites located at R1 and *ABI3* abundance in FTC and FTA samples (*r* = −0.6196 and *P* = 0.0002) (Figure 2C). These results suggest that methylation in the *ABI3* promoter is associated with transcriptional repression of *ABI3* in thyroid carcinoma tissues.

Interestingly sequencing analysis showed a C/T polymorphism (rs850526), which creates an additional CpG sites in *ABI3* R1 region in nearly 45,5% (5/11) of FTA and in 35,5% (6/17) of FTC samples (Supplementary Figure S2). To determine the methylation pattern, we estimated the contribution of all CpGs, including this unpredicted CpG site.

We found that 88% (8/9) of these CpG sites located at R1 were hypermethylated in FTCs compared to FTAs. However, these CpG sites were methylated to different degrees. These differences were largest at the CpG_4_, CpG_5_ and CpG_6_ sites (Figure 2D). Representative results of the bisulfite sequencing of 10 selected clones for a single patient with FTA and of 10 selected clones for a single patient with FTC is show in Figure 2E.

### Identification of a thyroid transcription factor-binding site in the promoter sequence of *ABI3* gene

**Figure 3:**
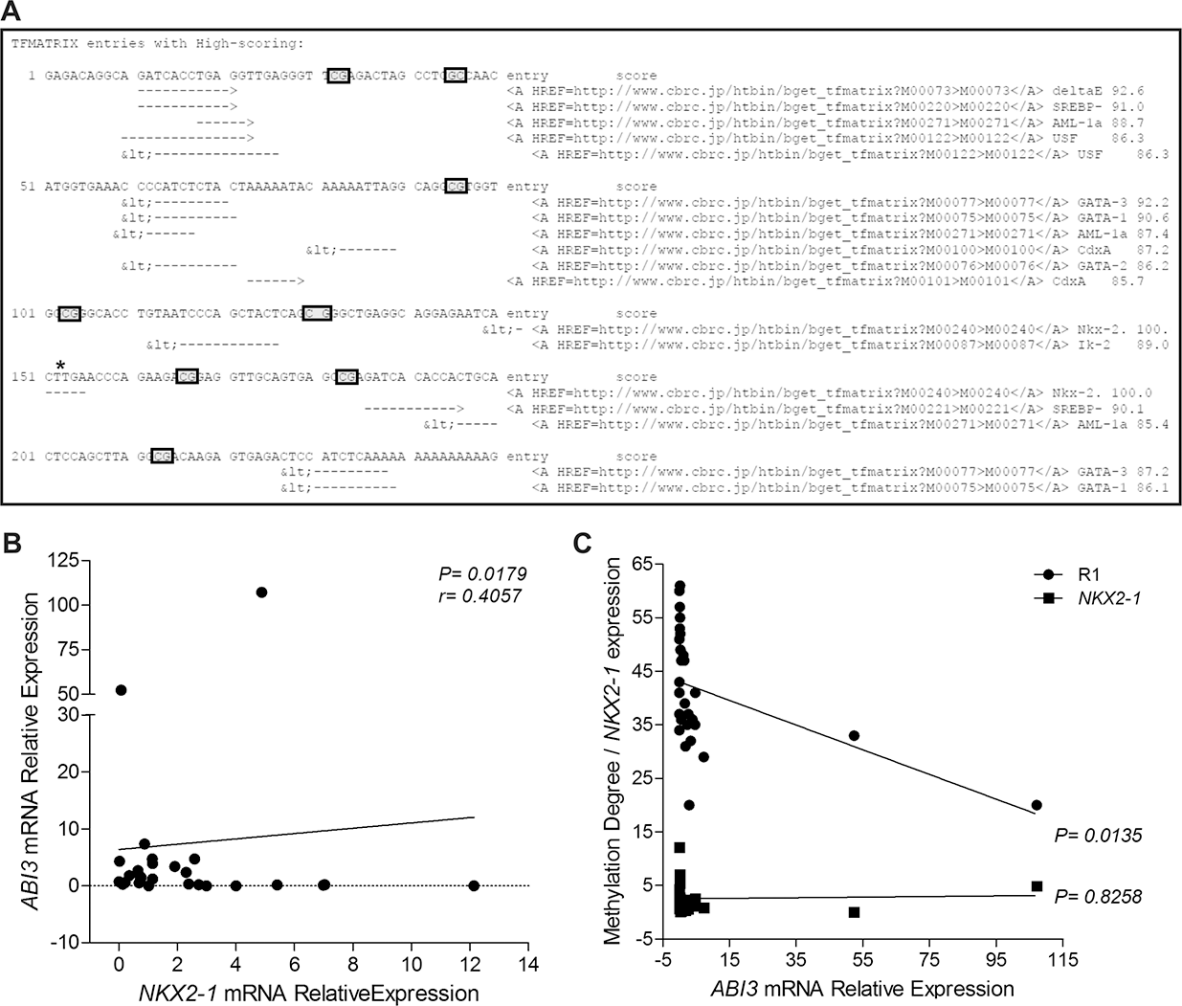
(**A**) *In silico* analysis of TFs sites within the R1 region of *ABI3* promoter. Asterisk indicates the NKX2-1 canonical binding site. (**B**) Positive correlation between *ABI3* and *NKX2-1* expression in FTA and FTC samples. (**C**) The linear regression analysis indicate that, although the expression of *NKX2-1* is required for the expression of *ABI3*, the degree of R1 methylation dictates the levels of *ABI3* expression in thyroid samples.

As hypermethylation of *ABI3* might occurs in a cancer- and tissue-specific manner, we search for putative transcription factors binding sites (TFBS) in the R1 region of *ABI3* gene. *In silico* analysis of this region identified a *NKX2-1* canonical binding site (CTTG) (Score 100.0) in the region flanked by the CpG_5_ and CpG_6_ sites. NKX2-1 encodes a protein initially identified as a thyroid-specific transcription factor (TTF1) (Figure 3A).

### *NKX2-1* expression and methylation degree of R1 region of *ABI3* gene are required to *ABI3* expression in thyroid cells

As lower expression of *NKX2-1* is correlated with progressive dedifferentiation of thyroid tumors, we next used qPCR to evaluate the expression levels of *NKX2-1* in thyroid samples and correlated *NKX2-1* and *ABI3* expression. Although the level of *NKX2-1* expression was comparable in thyroid tumors, a positive correlation was observed between *ABI3* and *NKX2-1* expression in thyroid tumors (*r* = 0.4057 and *P* = 0.0179) (Figure 3B). To investigate whether both *NKX2-1* expression and R1 methylation status affects the transcription of *ABI3*, we performed linear regression analysis. The linear regression analysis indicated that, although the expression of *NKX2-1* is required for the expression of *ABI3*, the degree of R1 methylation dictates the levels of *ABI3* expression in thyroid samples (R1 methylation: *P* = 0.0135; *NKX2-1* mRNA expression: *P* = 0.8258) (Figure 3C). These findings may explain why treatment with the demethylating agent 5-aza-dC did not restore *ABI3* expression in a non-thyroid carcinoma cell line (NPA).

**Figure 4:**
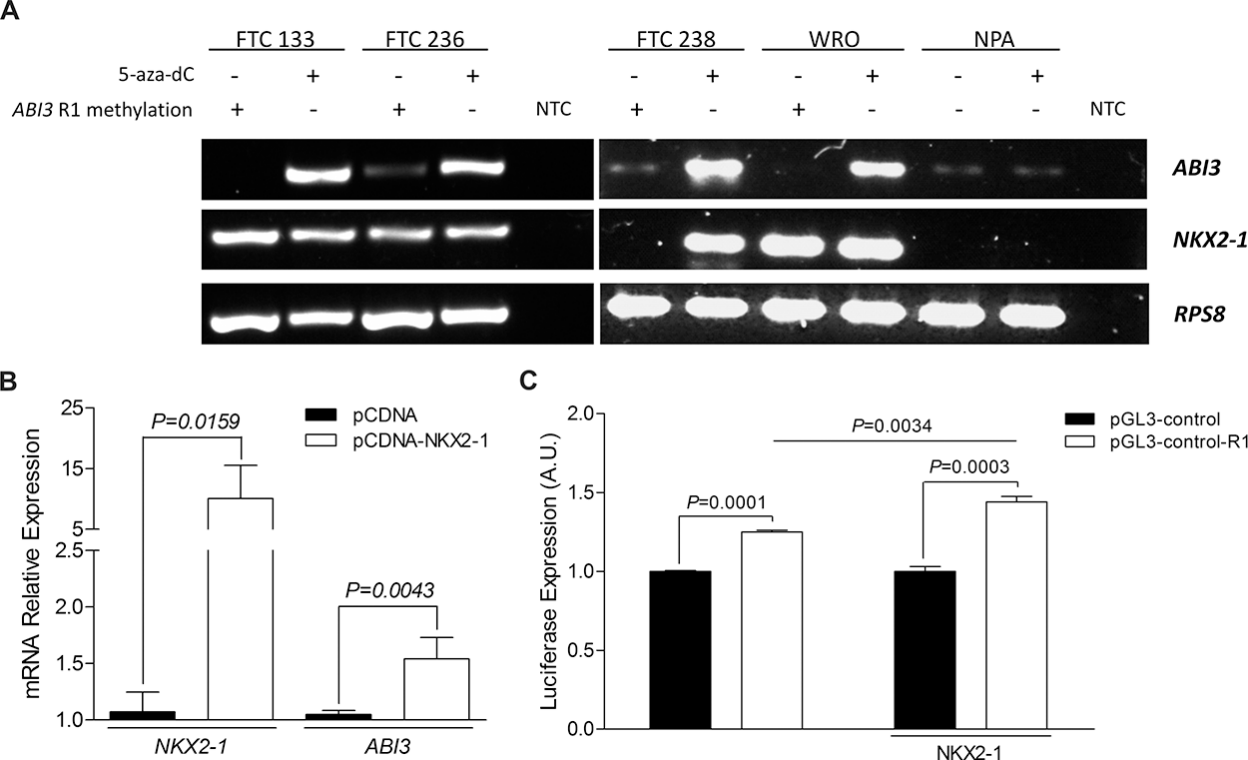
(**A**) Relationship between *NKX2-1* and R1 methylation in the *ABI3* expression in 5-aza-dC treated cells. *ABI3* expression was restored in follicular carcinoma cell lines (FTC 238, FTC 236, FTC 133 and WRO) when *NKX2-1* was present and R1 demethylated. In melanoma cells (NPA) the treatment with 5-aza-dC did not restore the expression of the *ABI3* in NPA cells. (**B**) Transient transfection of *NKX2-1* into NPA cells restored *ABI3* expression. (**C**) Luciferase reporter assays showing the regulatory effect of R1 region of *ABI3* promoter (white bars) compared to the pGL3-Control empty vector (black bars). Luciferase activity was increased after transfection of FTC 238 cells with pGL-control-R1 plasmid (white bars) as compared with cells transfected with pGL-control vector (black bars). The transcriptional activity was significantly increased when the cells were co-transfected with NKX2-1. NTC: no-template control.

To further validate the hypothesis that both *NKX2-1* expression and hypomethylation of R1 region of *ABI3* gene are required for *ABI3* expression, thyroid carcinoma and non-thyroid carcinoma cells were treated with demethylating-associated agent 5-aza-dC. The treatment with 5-aza-dC resulted in DNA demethylation at the R1 region in FTC 238 cells and the simultaneous restoration of *NKX2-1* and *ABI3* expression. In FTC 133, FTC 236 and WRO cells, which express endogenous *NKX2-1*, the treatment with 5-aza-dC resulted in restoration of *ABI3* expression. Although the CpG dinucleotides of R1 region of *ABI3* were hypomethylated in NPA cells, the treatment with 5-aza-dC did not restore the expression of the *ABI3* in NPA cells, most likely due to the fact that *NKX2-1* expression was not restored following treatment with 5-aza-dC (Figure 4A).

Having observed that *NKX2-1* is required for *ABI3* expression and that these CpGs sites at R1 region were hypomethylated in NPA cells, we next transiently transfected the pCDNA-NKX2-1 expression vector into NPA cells. Remarkable, the expression of *ABI3* was restored in NPA cells with ectopic expression of *NKX2-1*, while it was absent in NPA cells transfected with empty vector (*P* = 0.0043; Figure 4B).

### Functional analysis shows that R1 is a regulatory region in *ABI3* promoter

To measure the promoter activity of R1 region, the 312 pb fragment was cloned upstream of the luciferase reporter gene in the promoterless pGL3-Basic vector (designated pGL3-basic-R1). Luciferase activity was 0.011 ± 0.0013, 48 hours after co-transfection of pGL3-Basic-R1 and pRL-CMV and 0.007 ± 0.0071, 48 hours after co-transfection of pGL3-Basic empty vector and pRL-CMV, independently of *NKX2-1* expression (data not shown). Results were normalized to that of *Renilla* luciferase and presented in arbitrary units (AU) as mean ± SD.

Subsequently, the R1 fragment was cloned upstream the pGL3-Control containing SV40 promoter and enhancer. Using luciferase as reporter gene, the putative regulatory region of *ABI3* showed greater transcriptional activity compared to the pGL3-Control empty vector (*P* < 0.001; Figure 4C). The transcriptional activity was increased when the cells were co-transfected with NKX2-1 (*P* = 0.0034, Figure 4C). These findings suggested that R1 might act as a regulatory region of *ABI3* promoter and it might be modulated by NKX2-1.

## DISCUSSION

We previously reported that *ABI3* expression is lost in thyroid tumors and its re-expression in thyroid cells lines significantly suppresses cell growth *in vitro* and tumor growth *in vivo* [1]. In this study we investigated the hypothesis that *ABI3* is epigenetically silenced in thyroid carcinomas via DNA methylation.

Here we demonstrated that when thyroid carcinoma cell lines, which express virtually no *ABI3*, were grown in the presence of demethylating agent 5-aza-dC, the expression of *ABI3* was restored. On the other hand, the 5-aza-dC treatment did not induce the transcription of *ABI3* in a melanoma cell line. These findings were the first indication that methylation-induced transcriptional silencing of the *ABI3* in thyroid carcinomas and that this regulation might occur in a tissue-specific matter.

The next step was to determine the potential link between *ABI3* expression and methylation status of the 5 CpG sites located within intron 1 of the *ABI3* gene (designated IR), which was previously identified as methylated in leukemia [7]. Using bisulfite sequencing, we observed that the overall methylation status were quite similar between the 5-aza-dC treated and untreated thyroid carcinomas cell lines, providing indirect evidences that methylation beyond this CpG region could be associated with decrease or lack of *ABI3* expression in thyroid carcinomas cell lines and thyroid carcinoma samples.

As hypermethylation of gene-associated CpG islands are mostly linked to transcriptional silencing of tumor suppressor gene in cancer, we used *in silico* analysis to identify CpG islands spread over 10,000 pb upstream and 500 bp downstream of the translation start site of the *ABI3* gene. On the basis of the original criteria of Gardiner-Garden & Frommer [8] and Takai & Jones, there was no CpG island near or within the promoter region of *ABI3* gene.

Although DNA methylation occurs mainly in CpG-rich areas located near the promoters, is becoming clear that DNA methylation is dynamic and may occur in regions adjacent to the CpG-rich areas, which have a low density of CpG sites and named CpG shores. GpG shores exhibit tissue and cancer specific differential methylation. Beyond CpG island and shores, there are also regions of the genome with multiple adjacent CpG sites that show differential methylation patterns. These regions are classified as differentially methylated regions (DMR) and can occur in different contexts including cancer, denominated cancer-specific differentially methylated region (cDMR) [6].

We then extend our analysis by investigating the occurrence of CpG-rich regions, which did not meet the CpG island criteria, across the *ABI3* promoter region. We have limited the promoter region to 2498 base pairs upstream of the translation start site of the *ABI3*. *In silico* analysis showed three distinct regions (designated R1, R2 and R3) with multiple adjacent CpG sites within promoter region of *ABI3* gene. To ultimately quantitate total CpG methylation levels across each region before and after treatment with 5-aza-dC, these regions were submitted to bisulfite sequencing.

Although all regions showed some degree of methylation, the overall methylation status of R2 and R3 was not significantly different between 5-aza-dC treated and untreated cancer cells. However, statistical comparisons of the DNA methylation levels showed that the R1 was significant demethylated following 5-aza-dC treatment in FTC 238 and WRO thyroid carcinoma cells while there was no difference in the average degree of methylation in NPA cells (melanoma cells). We then evaluated the methylation status of these CpG sites in two additional follicular thyroid carcinoma cell lines (FTC 236 and FTC 133). Remarkable, drug-induced hypomethylation of R1 correlated with the reestablishment of *ABI3* expression in all follicular thyroid carcinoma cell lines. Importantly, we found a correlation between the degree of methylation and the level of *ABI3* expression. These findings further support the hypothesis that *ABI3* might be transcriptional silenced by methylation in thyroid tumors.

As cell lines might exhibit higher degree of methylation than the primary tumors they represent [9], we sought to specifically investigate the methylation degree of this candidate region in FTC, which essentially has no expression of *ABI3*, and in FTA samples, which expresses *ABI3* at high levels. Bisulfite sequencing of the DNA isolated from FTC samples also displayed a higher degree of DNA methylation within this region while FTAs shows a lower degree of methylation. Importantly, an inverse correlation was observed between *ABI3* expression and degree of methylation of CpG sites at the R1 of the *ABI3* promoter.

To estimate the degree of methylation at each CpG site, bisulfite-converted DNA was cloned and sequenced. This analysis quantitatively revealed the degree of methylation of each CpG site, including the non-predicted CpG_7_ in which the presence of an SNP generated a new CpG site. Most notably of all, the level of methylation at individual sites varied considerable. Three readable CpGs analyzed within this region (CpG_4_, CpG_5_ and CpG_6_) have increased methylation degree in FTCs (68%–73%) relative to that of FTAs (25%–48%). These differences were largest CpG_4_ and CpG_5_.

This study reveals a new region in the *ABI3* promoter that show differential methylation patterns in benign and malignant thyroid tumors. Methylation at some CpG sites was more strictly maintained in FTCs. It is possible that transcriptional silencing of *ABI3* in FTCs does not require hypermethylation of an entire CpG island but only few specific core CpG dinucleotides might be sufficient.

Although *ABI*3 was previously reported as methylated in a subset of patients with leukemia, our results differ from the previous study in which the five CpG sites, located within intron 1 of *ABI3* gene, were fully methylated and associated with decreased gene expression [7]. It's likely that the location of core regions and the degree of methylation required for *ABI3* silencing might vary according different tumors subtypes. Whether different genetic/epigenetic mechanism might be involved in the transcriptional regulation of *ABI3* in another cell type, it is still unclear.

Actually, differential methylation regions in cancer have been described in different tumor subtypes. Using next-generation sequencing, cancer-specific differentially methylated regions (cDMRs) were identified in prostate carcinomas [10].

As DNA methylation within regulatory regions may mediate gene silencing by causing interfering with the binding of transcription factor [11], we next focus on the identification of putative transcription factors binding sites at this differentially methylated region that would help to explain the tissue-specific expression of *ABI3*.

A closer look at the sequence configuration revealed the presence of one responsive element for the thyroid-specific transcription factors *NKX2-1* formerly called *TTF-1,* near the CpG_5_ and CpG_6_ sites. It is well known that *NKX2-1* plays an important role not only in tissue-specific gene expressions in adults as well as during development [12]. Interestingly, the expression of *NKX2-1* correlated with the expression of *ABI3* in thyroid samples. The linear regression analysis indicated that the high degree of methylation at R1 might be one event that leads to transcriptional inactivation of *ABI3* but seems that the *NKX2-1* input is also essential.

To functionally explore this hypothesis we initially investigated the levels of *ABI3* and *NKX2-1* in 5-aza-dC treated and untreated thyroid carcinoma and melanoma cells lines. We confirmed that *ABI3* was expressed in follicular carcinomas cells only when these specific CpG sites located at the promoter region of *ABI3* (R1 region) were demethylated and *NKX2-1* was present. In melanoma cells, even though the CpG sites at this specific region were hypomethylated, *ABI3* could not be detected, most likely because *NKX2-1* was absent in these cells. Conversely, ectopic expression of *NKX2-1* in melanoma cells lead to an increase in *ABI3* expression. The functional relevance of this region was further tested by luciferase dual transfection. Results of luciferase activity indicated that R1 might act as a regulatory region of *ABI3* promoter and it might be modulated by *NKX2-1*.

The mechanism by which methylation/demethylation occurs and how DNA methylation influences the binding of transcriptions factors (TFs) is still unclear. Recent studies challenge the conservative view that DNA methylation is the cause of altered gene expression. It has been suggested that the binding of the TFs triggers the loss of methylation prior to gene transcription [13]. Others have suggested that DNA methylation accumulates passively as a consequence of the absence of TF binding [14, 15]. If this hypothesis is correct, it is expected that methylation at transcription factor recognition sequences should be negatively correlated with transcription factor abundance.

From a more conventional point of view, epigenetic modifications influence the access of transcription factors to their binding sites. As example, cytosine methylation might directly disturb the affinity of transcription factors (TFs) towards their binding sites [15]. Finally, the occupation of regulatory region by methyl-CpG biding proteins can compete with transcription factor (TF) to its binding site, repressing the transcription [16].

Taken together, the results presented in this manuscript clearly indicate that mechanism governing *ABI3* expression in thyroid tumors involves methylation/demethylation at specific CpG sites located at the promoter region of *ABI3*, designed as cDMR. The results also uncovered a further layer in the regulation of the activation of *ABI3* promoter. We inferred that that DNA methylation helps to restrict *ABI3* activation when *NKX2-1* is expressed; *i.e.,* the presence of *NKX2-1* is needed for *ABI3* expression. Whether the binding of NKX2-1 to the *ABI3* promoter is sufficient to induce demethylation and, therefore, gene transcription or if the absence of NKX2-1 leads to cumulative methylation, needs further analysis.

## MATERIALS AND METHODS

### Thyroid samples

The series consists of 32 thyroid samples obtained from patients who underwent thyroid surgery at Hospital São Paulo (Universidade Federal de São Paulo). The study included 17 FTC and 11 FTA. Four normal thyroid tissues were used for expression analysis, as control group. The study was conducted under the approval of the Review Boards and Research Ethical Committee of the Universidade Federal de São Paulo.

### Cell lines

WRO (follicular thyroid carcinoma) and NPA (melanoma) cell lines were cultured in Dulbecco's modified essential medium (DMEM) supplemented with 10% fetal bovine serum (FBS) (Life Technologies, Grand Island, NY). NPA87 derivatives from M14/MDA-MB-435S melanoma cell line. FTC 238, FTC 236 and FTC 133 (follicular thyroid carcinoma cell line) were purchased from ECACC (European Collection of Cell Cultures, Cat. No 94060902, 06030202 and 94060901, respectively) and were cultured according manufacturer's recommended protocol. FTC 238 was culture in DMEM and Ham's F12 (1:1 mixture) supplemented with 5% FBS (FTC 238). FTC 133 was culture in DMEM and Ham's F12 (1:1 mixture) supplemented with 10% FBS. FTC 236 was culture in DMEM and Ham's F12 (1:1 mixture) supplemented with 10% FBS and 1 mU/mL TSH (Life Technologies) and 10 μg/mL insulin (Life Technologies).

### *In silico* analyses

Gene information was obtained from Ensembl Gene ID ENSG00000108798 and NCBI NC_000017.11. The sequences from 10,000 pb upstream and 500 bp downstream of *ABI3* translational start codon (ATG +1), which included intron 1 and exon 1 of *ABI3* gene, were extracted and the detection of the CpG islands was performed using Methyl Primer Express Software (Applied Biosystems) according to the criteria established by Gardiner-Garden & Frommer [8] and Takai & Jones [17] *i.e*., a CpG island is defined as a region of at least 200 pb, with proportion of GC content greater than 50% and observed to expected (O/E) CpG ratio ≥ 0.6. The analysis also focused on the presence of CpG-rich regions that did not meet the CpG island criteria at the putative promoter of *ABI3* gene (−2498 pb to −498 pb relative to ATG +1).

### 5-aza-2′-deoxycytidine treatment

For demethylation analysis, cell lines (3 × 10^5^) were cultured in 35-mm culture plates and treated with 15 μM of demethylating agent 5-aza-dC (Sigma-Aldrich, St. Louis, MO) diluted in dimethylsulfoxide (DMSO) or vehicle only (untreated cells) for a total of 72 h as previously described [18]. Fresh media containing either DMSO or 5-aza-dC were changed once every 24 h. Drug treatment (5-aza-dC) experiments were performed in triplicate.

### Total RNA isolation and quantitative RT-PCR (qPCR)

Total RNA was isolated from cell lines and thyroid samples using TRIzol reagent according to the manufacturer's recommendation (Invitrogen, Life Technologies) and quantitated using a NanoDrop spectrophotometer. Total RNA (1 μg) was treated with DNAse (Ambion, Life Technologies) and reverse transcribed to cDNA using Super-Script III Reverse Transcriptase kit with an oligo(dT)_12–18_ primer and 10 units of RNase inhibitor (Invitrogen), according to the manufacturers’ recommended protocols. An aliquot (1 μL) of cDNA was used in a 12 μL PCR reaction containing SYBR Green PCR Master Mix (Applied Biosystems, Life Technologies) and 3, 2 pmol of each specific primer for the target genes (*ABI3 and NKX2-1*) or reference gene (*RPS8*). The PCR reactions were performed in triplicate, and the Ct was obtained using Applied Biosystem software and averaged (SD < 1.0). The relative expression (RE) was calculated according to the comparative ΔΔCt method. RE values in thyroid tumor samples were normalized to the *RPS8* and then to the normal thyroid tissues. RE values in cell lines was normalized to the RPS8 and then to the vehicle-treated control cells or empty vector transfected cells. The PCR primers and conditions are summarized in Supplementary Table S1.

### DNA isolation and bisulfite sequencing

For methylation analyses, DNA was purified from untreated or 5-aza-dCt treated cells or thyroid samples as previously reported [19] and quantitated by absorbance at 260 nm. To deaminate unmethylated cytosines, and thus distinguish methylated from unmethylated alleles, DNA (1 μg) was converted using EpiTect Bisulfite conversion kit (QIAgen, Hilden, Germany) according to the manufacturer's instructions. Bisulfite-converted DNA (10–30 ng) was used as template in a 25-μL PCR reactions containing 10 mM dNTPs, 50 mM MgCl_2,_ 20 mM Tris-HCl, 50 mM KCl, 1,5U Platinum Taq DNA polymerase (Life Technologies) and 10 pmol of specific primers. Primers flanking the CpG sites located within intron1 (designed IR) or within the promoter region of *ABI3* (designed R1, R2 and R3) were designed (Figure 1B). Primers sequence and PCR conditions are described in Supplementary Table S1.

PCR products were then cloned into pCR2.1-TOPO vector using TOPO TA Cloning kit (Life Technologies) according to the manufacturer's instructions. Nearly 8–12 independent colonies were picked randomly and the inserts were amplified and sequenced using BigDye Terminator v3.1 Cycle Sequencing Kit (Applied Biosystems). After bisulfite treatment, the degree of methylation at each CpG site in the individual regions were determined from the ratio of methylated cytosines to total cytosines and expressed as percentage.

### Prediction of TFBS

As gene expression is primarily regulated by TFs, we search for putative TFBS in the promoter sequence of *ABI3* gene using FT Search software available in http://diyhpl.us/~bryan/irc/protocol-online/protocol-cache/TFSEARCH.html. The threshold score was 85.0 (default).

### Transient transfection of NKX2.1 into NPA cells

pcDNA-NKX2.1 [18] or empty vector were transiently transfected into NPA cells (5 × 10^5^) using Lipofectamine LTX with Plus Reagent (Life Technologies) according to the manufacture's instructions. Forty-eight hours post-transfection, the cells were harvested and assayed for *ABI3* and *NKX2-1* expression. To this end, total RNA isolation, cDNA synthesis and qPCR were performed as above. Assays were performed in triplicate. The RE was calculated as above-mentioned.

### Luciferase reporter constructs

We functionally evaluated the efficacy of regulatory sequence (R1) of *ABI3* by analysis of luciferase expression. The region (R1) located at −2467 pb to −2285 pb relative to *ABI3* translational start codon (ATG +1) was PCR amplified from DNA isolated from a normal thyroid tissue. PCR primers were designed to include restriction sites. The *Kpn*1 restriction site was add in the primer sense and *Xho*I site in the antisense primer (Supplementary Table S1). The PCR-amplified product was digested with *Kpn*I and *Xho*I restriction enzymes (New England Biolabs, Hitchin, UK) and directionally cloned into the same restriction sites upstream to a promoterless firefly luciferase gene of the pGL3-Basic vector and into the pGL3-Control (Promega, Madison, WI), which contains a luciferase gene driven by the SV40 promoter. The empty pGL3-Basic and pGL3-Control were used as negative control. Mach1 competent bacteria strain was transformed with the resulting constructs (designed pGL3-Basic-R1 or pGL3-Control-R1) by the heat shock method. The constructs were isolated and sequenced using BigDye Terminator v3.1 Cycle Sequencing Kit (Applied Biosystems).

### Transient transfection and luciferase assays

FTC 238 cells (4 × 10^4^) were co-transfected with 500 ng of the firefly luciferase constructs (pGL3-Basic-R1, pGL3-Control-R1, empty pGL3-Basic or empty pGL3-Control) and 10 ng of *Renilla* luciferase reporter vector (pRL-CMV) using Lipofectamine LTX with Plus Reagent (Life Technologies), according to the manufacture's instructions. The pRL-CMV (Promega), which contains the *Renilla* luciferase gene driven by the CMV enhancer and early promoter, was used to normalize for transfection efficiency. All transfections were performed in the presence or absence of pcDNA-NKX2-1 vector (320 ng), which expresses the transcription factor NKX2-1 [18].

Forty-eight hours post-transfections, firefly and *Renilla* luciferase activities were measured in cell lysates using Dual Luciferase Assay System (Promega) with the Victor3 spectrophotometer (Perkin Elmer, Massachusetts, USA). The relative activity of firefly luciferase was standardized to the *Renilla* luciferase control and results expressed in arbitrary units (AU). Luciferase assays were performed in triplicate.

### Statistical analysis

Statistical analyses were performed using GraphPad Prism v5.01 Software (GraphPad Software). Shapiro-Wilk test was used to verify the normality of distribution. Comparisons between two groups were performed using Student *t* test (when demonstrate normal distribution) or Mann-Whitney test (non-normal distribution). For contingency analysis, Fisher's exact test was employed. Pearson correlation and multiple linear regression analysis were performed. The results were expressed as mean ± SD. The results with *P* < 0.05 were considered statistically significant.

## SUPPLEMENTARY MATERIALS FIGURES AND TABLES


