# Supplementary material for: Transcriptional regulation of the potential tumor suppressor ABI3 gene in thyroid carcinomas: interplay between methylation and NKX2-1 availability
Source: Oncotarget. 2016 Mar 27;7(18):25960–70. doi: 10.18632/oncotarget.8416 (PMC5041957; doi:10.18632/oncotarget.8416)
Supplement: Supplementary file 1 [file oncotarget-07-25960-s001.pdf]

Transcriptional regulation of the potential tumor suppressor *ABI3* gene in thyroid carcinomas: interplay between methylation and *NKX2-1* availability

Supplementary Materials

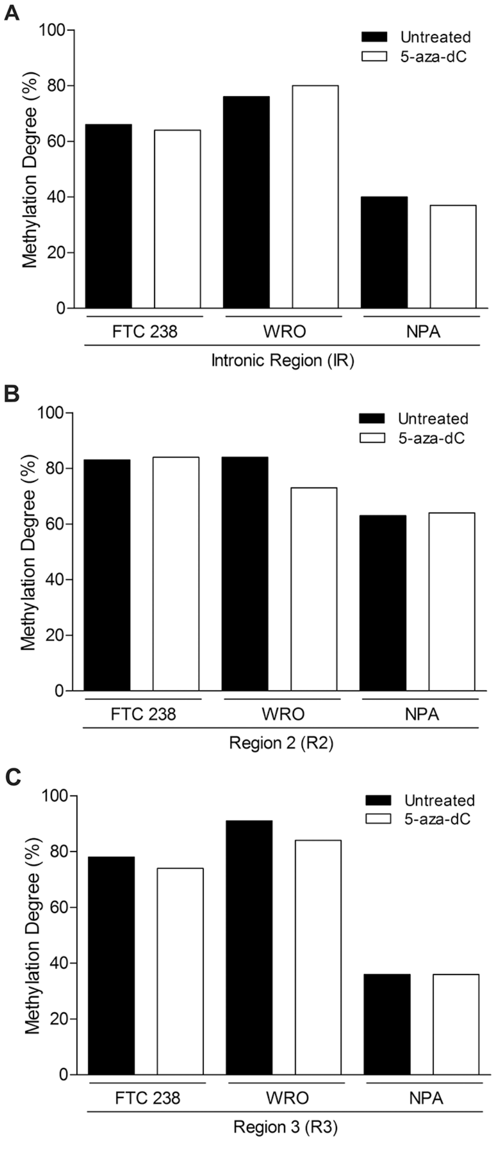

Supplementary Figure S1: Methylation degree of IR (A), R2 (B) and R3 (C) in untreated and 5-aza-dC treated follicular carcinoma cells (FTC 238 and WRO) and melanoma cells (NPA).

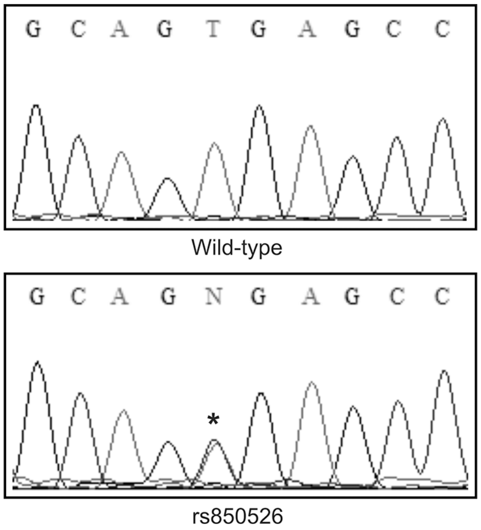

Supplementary Figure S2: Electropherogram showing the wild type sequence and the SNP rs850526 (*), which created a new CpG site within R1 of the *ABI3* promoter.

Supplementary Table S1: Primers sequences, PCR conditions and product sizes

Gene	Sequence (5′–3′)	Application	Annealing Temp (°C)	Primer [Final][a]	Expected Product Size (pb)
ABI3	Forward: TAAGCACGCTGGGCCAGAT	Expression	60	3,2	166
	Reverse: CAGCCAAAGTTGAGGGGTCT				
NKX2-1	Forward: AATGGGTTTTCCACGCTAGG	Expression	60	3,2	93
	Reverse: TGCCCAGAGTGAAGTTTGGT				
RPS8	Forward: AACAAGAAATACCGTGCCC	Expression	60	6,4	125
	Reverse: GTACGAACCAGCTCGTTATTA				
ABI3 R1	Forward: ATAGGTAGATTATTTGAGGTTGAG	BSF[b]	53	10	264
	Reverse: CTTATCTCTCTCTCTCTCTTTT				
ABI3 R2	Forward: TGATGTTTAAGTTGATTATAGGTT	BSF	53	10	494
	Reverse: AAACAAAAACATTAAAAATAACCA				
ABI3 R3	Forward: TGATATTTATTTAATGATAAGGGAT	BSF	53	10	681
	Reverse: AAAACTTCCTTAAAAAACTCAAAA				
ABI3 IR	Forward: TTAGAGATGTTTGGGATAGATAGTG	BSF	53	10	177
	Reverse: AAAAAATTCTTCTAAAACCTCACCC				
ABI3 R1	Forward: ACTGTGGCTCATGCCTGTAATC	Sequencing	63	10	312
	Reverse: ACCCCCTTGTCTCTCTCTCTCT				
ABI3 R1	Forward: CCGGGTACCACTGTGGCTCATGCCTGTAATC	Cloning	63	10	330
	Reverse: CCGCTCGAGACCCCCTTGTCTCTCTCTCTCT				

[a]pmol.
[b]BSF; bisulfite converted sequence.
